# Smartphone addiction, emotional coping strategies, and mental health symptoms in Chinese university students: a gender-based analysis

**DOI:** 10.3389/fpsyg.2026.1833715

**Published:** 2026-07-22

**Authors:** Xu Liu, Zhenggui Gu, Lulu Shi

**Affiliations:** 1 School of Educational Sciences, Xinxiang University, Xinxiang, Henan, China; 2 Department of Human Development and Family Studies, Faculty of Human Ecology, Universiti Putra Malaysia, Serdang, Selangor, Malaysia; 3 School of Music, Xinxiang University, Xinxiang, Henan, China; 4 School of Education, Weifang University of Science and Technology, Weifang, Shandong, China

**Keywords:** emotional coping strategies, gender differences, mental health symptoms, PLS-SEM, smartphone addiction

## Abstract

**Background:**

Smartphone addiction is an increasing mental health concern among university students, yet the emotional coping correlates related to this association remain insufficiently understood. This issue is strategically important in Chinese higher education because university students represent a large digitally embedded population facing academic, developmental, and psychosocial pressures. This study examined the cross-sectional associations among elevated addiction-like smartphone use, emotional coping strategies, mental health symptoms, and gender among Chinese university students who met the SAS-SV screening threshold.

**Methods:**

Guided by stress-coping theory, this study used convenience recruitment followed by criterion-based screening. A total of 2,401 students from two public universities in China completed the Smartphone Addiction Scale-Short Version (SAS-SV) screening procedure, and 1,276 students who met the SAS-SV screening threshold were included in the final analysis. Elevated addiction-like smartphone use was assessed using the SAS-SV, mental health symptoms using the Kessler Psychological Distress Scale (K10), and emotional coping strategies using the Emotion Regulation Questionnaire (ERQ), including reinterpretation strategy and suppression strategy. Direct and indirect statistical associations were tested using partial least squares structural equation modelling (PLS-SEM). Measurement invariance was evaluated before gender-based multigroup analysis.

**Results:**

Smartphone addiction was positively associated with mental health symptoms (*β* = 0.748, *t* = 55.524, *p* < 0.001), suppression strategy (*β* = 0.357, *t* = 12.075, *p* < 0.001), and reinterpretation strategy (*β* = 0.214, *t* = 7.230, *p* < 0.001). Suppression strategy showed a positive indirect association between smartphone addiction and mental health symptoms, whereas reinterpretation strategy showed a negative indirect association. The model explained 66.2% of the variance in mental health symptoms. Gender did not significantly moderate the direct pathway, but the indirect pathway through reinterpretation strategy was significant among male students only.

**Conclusion:**

The findings identify theoretically meaningful cross-sectional associations among smartphone addiction, emotional coping strategies, and mental health symptoms. Suppression and reinterpretation strategies showed different patterns of association with mental health symptoms, suggesting that emotional coping strategies may be relevant targets for future longitudinal and intervention research. As the data were collected at a single time point, the observed relationships cannot support causal inference and should instead be interpreted as associations.

## Introduction

The rapid proliferation of smartphones has profoundly transformed daily life, particularly among university students, for whom smartphones have become indispensable tools for communication, learning, and social interaction (Giansanti, 2025; Zhu et al., 2025). Smartphone addiction and problematic smartphone use are related, but conceptually distinct: problematic smartphone use is a broader term referring to maladaptive or uncontrolled smartphone engagement, whereas smartphone addiction denotes a more specific addiction-like pattern involving impaired control, salience, withdrawal-like discomfort, tolerance-like escalation, and functional interference. This distinction is considered further in the literature review. Recent evidence indicates that smartphone addiction is common among Chinese university students. For example, a meta-analysis of 67 Chinese studies involving 129,996 college students estimated a pooled prevalence of 28.49%, with reported rates ranging from 4.05 to 83.60% across studies (Wang and Abdullah, 2026). A study of Chinese medical college students also reported a prevalence of 49.5% (Zhang et al., 2024). These findings suggest that addiction-like smartphone use is not a marginal phenomenon but a substantial concern in Chinese higher education. A growing body of evidence further indicates that smartphone addiction is associated with adverse psychological outcomes, including anxiety, depression, stress, and overall mental health symptoms (Luo et al., 2025; Xu et al., 2025; Li et al., 2025).

### Smartphone addiction and mental health symptoms

University students represent a particularly vulnerable population for elevated addiction-like smartphone use because they experience academic pressure, developmental transitions, and increased autonomy in technology use (Giansanti, 2025). Empirical studies have repeatedly shown that individuals with higher levels of smartphone addiction scores tend to report greater psychological difficulties, including depression, anxiety, and emotional exhaustion (Lei et al., 2020; Feng and Dou, 2024; Kong et al., 2025). Conceptually, addiction-like smartphone use may be interpreted as a chronic digital stress context because excessive or uncontrolled smartphone engagement can expose students to persistent cognitive, emotional, and behavioral demands rather than to a single acute stress event. Constant connectivity may be associated with attentional fragmentation and perceived obligation to remain available; information overload may be associated with cognitive strain; sleep disruption may reduce recovery capacity; and repeated social comparison or fear of missing out may co-occur with negative affect. These processes may accumulate across daily academic and interpersonal contexts and may therefore be statistically linked to stress-related arousal and mental health symptoms (Koban et al., 2023; Ndayambaje and Okereke, 2025).

However, existing studies have largely focused on direct associations between smartphone addiction scores and mental health symptoms, offering limited insight into the emotional coping correlates that may be involved in this relationship. Identifying such associations is valuable for advancing theory and informing future longitudinal and intervention research.

### Emotional coping strategies as statistical indirect correlates

Drawing on Stress-Coping Theory (Lazarus and Folkman, 1984), psychological responses to stress are shaped not only by exposure to stressors but also by how individuals appraise and manage emotionally demanding experiences. Lazarus and Folkman’s framework distinguishes problem-focused coping, which targets the source of stress, from emotion-focused coping, which regulates emotional responses to stress. The present study focuses on emotion-focused coping because the key outcome is mental health symptoms and because smartphone addiction is often experienced as a persistent and difficult-to-eliminate behavioral pattern in daily student life. In this context, students may not immediately remove the stressor itself; instead, their emotional responses to smartphone-related stress may be regulated through strategies such as reinterpretation or suppression.

Problem-focused coping was not included as a separate variable because the study was designed to examine emotional coping correlates that are theoretically proximal to anxiety, depressive symptoms, fatigue, and distress as assessed by the Kessler Psychological Distress Scale (K10). Including problem-focused coping would require a broader behavioral coping model and additional measures of active problem solving, planning, and instrumental support, which were beyond the scope of the present study. This scope has been clarified to avoid implying that problem-focused coping is unimportant; rather, it represents a complementary direction for future research.

Emotional coping strategies are techniques aimed at managing affective responses associated with stress and adversity (Gross, 1998). Within the emotion regulation literature, cognitive reinterpretation and expressive suppression represent two theoretically distinct regulatory processes. Reinterpretation modifies the appraisal of an emotion-eliciting event before the emotional response is fully generated, whereas suppression attempts to control the observable expression of emotion after the response has already been activated. Accumulated evidence suggests that these two strategies are differentially related to psychological well-being, with reinterpretation generally reflecting an adaptive regulatory pattern and suppression showing stronger links with psychological maladjustment and heightened mental health symptoms (Eisma et al., 2023; Toh et al., 2024; Gross and John, 2003).

In the context of elevated addiction-like smartphone use, students who rely heavily on smartphones in emotionally demanding situations may differ in their use of adaptive and maladaptive coping strategies. For example, problematic smartphone use has been associated with digital stress and emotion regulation difficulties (Zhan et al., 2025). Although emerging studies suggest that emotional coping strategies are relevant to technology-related mental health outcomes (Garzon Umerenkova et al., 2026; Ahmed and Shaban, 2026), empirical research examining statistical indirect associations linking smartphone addiction scores, emotional coping strategies, and mental health symptoms remains limited, particularly among Chinese university students.

### The moderating role of gender

Gender differences may further shape the associations among smartphone addiction, emotional coping strategies, and mental health symptoms. Prior research has documented systematic gender differences in emotional experience, regulation strategies, and psychological adjustment. Females tend to report higher levels of mental health symptoms and greater use of cognitive reappraisal, whereas males are more likely to rely on expressive suppression (Wang et al., 2022; Preston et al., 2022).

Prior studies have reported gender-based variation in smartphone-use behaviors and risk for problematic use, suggesting that smartphone addiction may be associated with psychological outcomes through gender-specific patterns (Tu et al., 2023; Wei et al., 2024). From the perspective of stress and coping, gender may function as a moderating context in which the direct and mediated associations among smartphone addiction, emotion regulation strategies, and mental health symptoms differ in strength. However, empirical evidence testing gender-based differences in these associations remains scarce.

### The present study

**Figure 1:**
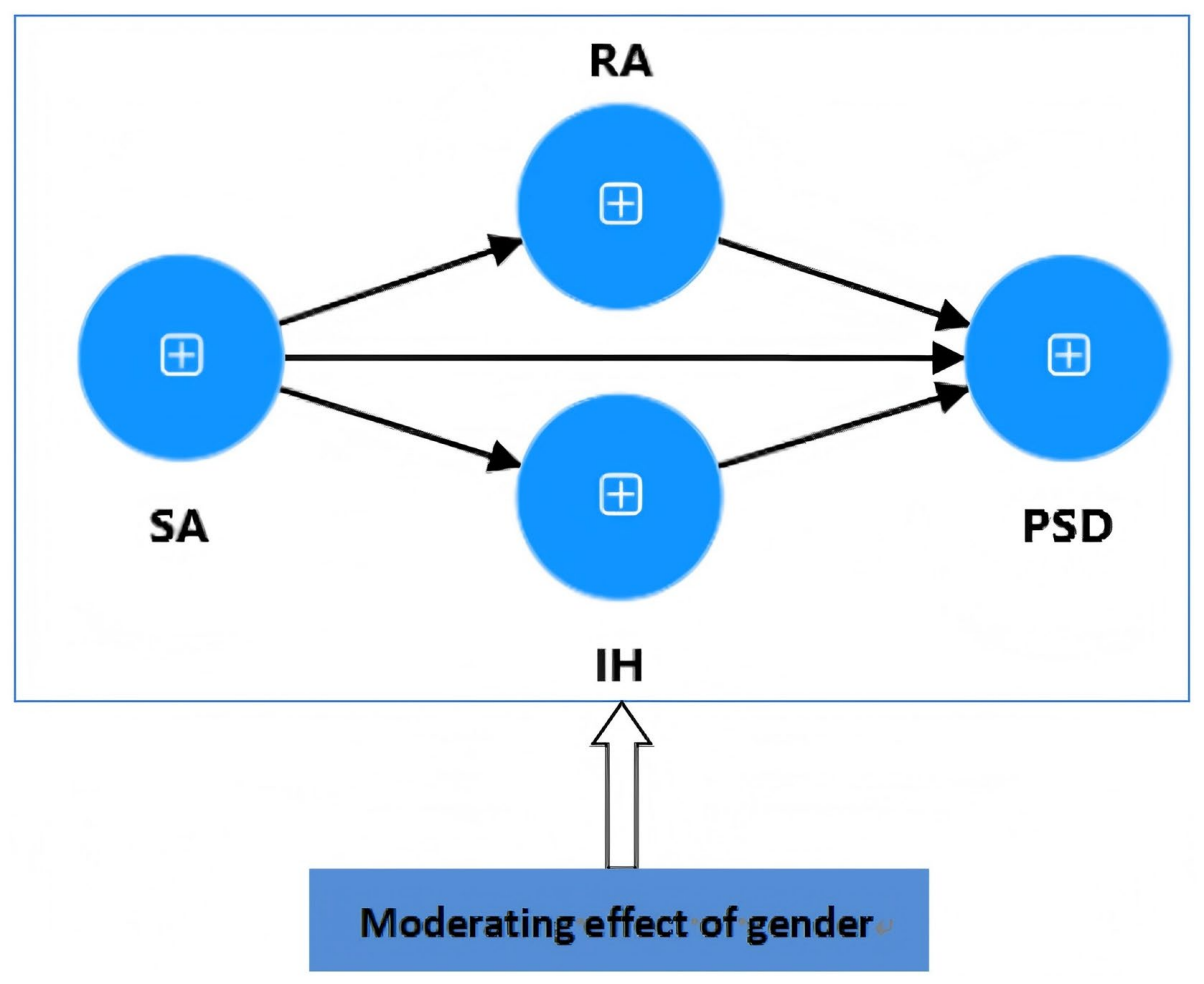
The theoretical model. SA, smartphone addiction; RA, reinterpretation strategy; IH, suppression strategy; PSD, mental health symptoms.

To advance the existing literature, this study developed an integrated analytical model to clarify how smartphone addiction scores are associated with mental health symptoms among Chinese university students who met the SAS-SV screening threshold (see Figure 1). In this model, emotional coping strategies were examined as indirect statistical correlates, and gender was used as a grouping variable to assess whether the hypothesised pathways differed across male and female students. Specifically, this study aimed to (a) test the direct association between smartphone addiction scores and mental health symptoms, (b) test whether reinterpretation and suppression strategies are involved in statistical indirect associations between smartphone addiction scores and mental health symptoms, and (c) explore whether these direct and indirect associations differ by gender. By integrating Stress-Coping Theory with emotional coping strategy research, this study seeks to provide a more precise account of the cross-sectional associations linking elevated addiction-like smartphone use, emotional coping, and mental health symptoms in university settings.

## Method

### Study framework and analytical strategy

The present investigation adopted a quantitative cross-sectional framework to examine the associations linking smartphone addiction scores, emotional coping strategies, and mental health symptoms among Chinese university students. The conceptual model was grounded in Stress-Coping Theory and specified reinterpretation and suppression strategies as statistical indirect correlates. Gender was examined as a grouping variable to determine whether structural associations varied across male and female students. Given the cross-sectional design, the estimated paths were treated as statistical associations and should not be interpreted as causal effects or temporal sequences. Data were analysed using partial least squares structural equation modelling (PLS-SEM) in SmartPLS 4.0. PLS-SEM was used as an acceptable variance-based SEM approach for estimating latent-variable associations, indirect statistical pathways, and gender-based multigroup differences within the specified analytical model (Hair et al., 2019; Shmueli et al., 2019). The use of PLS-SEM should not be interpreted as indicating that covariance-based SEM would be inappropriate; rather, PLS-SEM was selected as one defensible analytical option aligned with the study’s emphasis on explained variance, predictive relevance, and comparison of structural associations across groups. The large sample size increased the stability of parameter estimates and did not preclude the use of alternative SEM approaches. Therefore, the results are interpreted as variance-based estimates of theoretically specified statistical associations, not as evidence of absolute model fit or causal pathways.

### Participants and data collection procedure

This study received ethical approval from the Ethics Committee for Research Involving Human Subjects at Universiti Putra Malaysia on February 16, 2024 (Reference No.: JKEUPM-2023-1426). The study protocol submitted for ethical review specified recruitment and questionnaire-based data collection at the participating universities in China. Administrative permission to conduct the survey was also obtained from Xinxiang Medical University and Xinxiang University before recruitment. These institutional permissions authorised classroom-based recruitment and on-site data collection at the participating Chinese universities. All participants provided written informed consent, which was documented through paper-based questionnaires prior to participation.

Participants were undergraduate students recruited from Xinxiang Medical University and Xinxiang University in China in April 2024. Data were collected through paper-based questionnaires administered in classroom settings. Trained research assistants distributed the questionnaires on site, explained the purpose of the study, informed students that participation was voluntary and anonymous, and collected the completed questionnaires immediately after completion. The study used convenience recruitment followed by criterion-based screening because the aim was not to estimate the population prevalence of smartphone addiction but to examine theoretically guided associations among students with elevated addiction-like smartphone use.

As part of the criterion-based screening procedure, all interested students (*N* = 2,401) first completed the Smartphone Addiction Scale-Short Version (SAS-SV) as a standardized screening instrument. The SAS-SV was used as an *a priori* pre-selection tool, meaning that eligibility for the final analytical sample was determined before hypothesis testing and model estimation. Following the gender-specific cutoff criteria proposed in the original SAS-SV validation study, students scoring 33 or above for females and 31 or above for males were classified as meeting the SAS-SV screening threshold for elevated addiction-like smartphone use (Kwon et al., 2013). The Chinese version of the SAS-SV has demonstrated acceptable reliability and factorial validity among Chinese college students (Zhao et al., 2022).

The participant flow was as follows. A total of 2,401 undergraduate students completed the initial SAS-SV screening procedure sufficiently for eligibility determination. Of these students, 1,125 scored below the gender-specific SAS-SV cutoff values and were therefore not included in the analytical sample. The remaining 1,276 students met the SAS-SV screening threshold and constituted the final analytical sample (*N* = 1,276). The cutoff-based classification was used only for screening and sample definition; it should not be interpreted as a clinical diagnosis of smartphone addiction.

A total of 1,276 Chinese university students were included in the final analysis. Participants ranged in age from 18 to 24 years, and the mean age was 19.94 years. SPSS 27.0 was used to conduct preliminary data screening and descriptive analyses. Screening included checks for missing values, response range errors, incomplete questionnaires, invalid response patterns, descriptive statistics, and distributional properties. Item-level missingness was minimal across all scales, with missing responses accounting for less than 1% of the total item responses. Questionnaires with substantial missing information or invalid response patterns were not retained for analysis. Sporadic missingness was distinguished from substantial missingness at the scale level: missingness was treated as sporadic when only isolated item responses were missing within a scale, and sufficient valid items remained to compute a scale score, whereas questionnaires with extensive missing information across a scale or invalid response patterns were treated as substantial missingness. Remaining sporadic missing values were handled using scale-mean replacement because the proportion of missing data was low and because this approach preserves cases when item-level missingness is minimal. Hypothesis testing and model evaluation were conducted using SmartPLS 4.0.

### Instruments

#### Smartphone addiction

The Smartphone Addiction Scale–Short Version (SAS-SV; Kwon et al., 2013) was used to assess elevated addiction-like smartphone use. The instrument consists of 10 items scored on a 6-point Likert scale from strong disagreement to strong agreement, with higher scores representing greater smartphone addiction severity. Gender-specific cutoff values were used only for screening classification, with scores of 31 or higher for male students and 33 or higher for female students indicating that participants met the SAS-SV screening threshold. In this study, the measure showed excellent reliability, with Cronbach’s *α* = 0.896.

#### Mental health symptoms

Mental health symptoms were measured using the Kessler Psychological Distress Scale (K10; Kessler et al., 1994), which assesses anxiety, fatigue, and depressive symptoms experienced over the previous month. The scale consists of 10 items rated on a 5-point frequency scale. Higher total scores reflect greater mental health symptoms (Zhou et al., 2008). Internal consistency in the current sample was high, with Cronbach’s alpha = 0.935.

#### Emotional coping strategies

Emotional coping strategies were measured using items assessing reinterpretation strategy and suppression strategy, based on established emotion regulation research (Gross and John, 2003; Wang et al., 2022). Responses were recorded on a 7-point Likert scale. Reliability analyses indicated satisfactory internal consistency for both subscales, with alpha coefficients of 0.878 for reinterpretation strategy and 0.842 for suppression strategy.

### Measurement invariance and multigroup procedure

Measurement invariance was assessed before comparing structural paths across gender groups. This was done using the measurement invariance of composite models (MICOM) procedure (Henseler et al., 2016), which comprises three sequential tests: configural invariance, compositional invariance, and equality of composite means and variances. Configural invariance was evaluated by confirming that the male and female models used the same indicators, data-treatment procedures, and algorithm settings. Compositional invariance was examined through permutation testing, with support indicated when the original composite correlation was not significantly lower than the permutation-based reference value. The equality of composite means and variances was then assessed using permutation-based confidence intervals. Partial measurement invariance was regarded as sufficient for interpreting gender differences in structural path coefficients.

After measurement invariance was established, gender-based PLS multigroup analysis was conducted. Direct and indirect associations were estimated separately for male and female students, and between-group differences were evaluated using PLS-MGA. Gender was therefore treated as a grouping variable rather than as a continuous moderator.

### Data analysis

Preliminary analyses were conducted in SPSS 27.0 to inspect missing values, response range errors, invalid response patterns, descriptive statistics, and bivariate Pearson correlations. Harman’s single-factor test was used as an initial check for common method variance. The measurement and structural models were then estimated in SmartPLS 4.0 using the PLS algorithm, with statistical significance tested through nonparametric bootstrapping with 5,000 resamples.

Following PLS-SEM reporting recommendations (Hair et al., 2019), the reflective measurement model was evaluated in terms of indicator loadings, Cronbach’s alpha, composite reliability, average variance extracted (AVE), and discriminant validity using the heterotrait–monotrait ratio of correlations (HTMT). Structural model assessment included variance inflation factors (VIF) for collinearity, standardized path coefficients, t values, *p* values, 95% confidence intervals, *R*^2^ values, *f*^2^ effect sizes, and *Q*^2^_predict for predictive relevance. Because the primary analysis used PLS-SEM, model adequacy was summarized using reliability, validity, explanatory power, collinearity, effect size, and predictive relevance indices. The magnitude of *f*^2^ was classified according to conventional benchmarks, with values near 0.02, 0.15, and 0.35 indicating small, medium, and large effects, respectively.

## Results

### Preliminary data screening and common method bias

Before hypothesis testing, the dataset was screened in SPSS 27.0 for missing data, response-range violations, invalid response patterns, and descriptive distributional features. Potential common method variance was assessed using Harman’s single-factor analysis. The first unrotated factor accounted for 48.406% of the total variance, which was below the conventional 50% criterion, suggesting that no single factor exerted a dominant influence on the covariance structure. Collinearity-related bias was further examined through variance inflation factors (VIFs) in the structural model, with values ranging from 1.000 to 1.244 and remaining below commonly accepted thresholds. Alongside procedural safeguards such as anonymous responding and standardized administration, these results indicated that common method bias was unlikely to fully explain the observed associations. Nonetheless, given that all variables were measured through self-report instruments at a single time point, possible common method variance was retained as a methodological limitation.

### Sample characteristics and descriptive statistics

The final sample included 1,276 students, comprising 639 female students (50.1%) and 637 male students (49.9%), indicating a nearly balanced gender distribution. Participants ranged in age from 18 to 24 years, with a mean age of 19.94 years (SD = 1.34). Most participants were aged 20–21 years (49.3%), followed by those aged 18–19 years (38.2%). Regarding first smartphone ownership, more than half of the participants reported obtaining their first smartphone between ages 16 and 21 years (53.3%), followed by those who first owned a smartphone between ages 11 and 15 years (41.9%). The mean age of first smartphone ownership was 15.39 years (SD = 2.51). Monthly household income was most commonly reported in the CNY 2,684–4,184 range (35.4%), followed by the CNY 4,185–7,920 range (32.5%), with a median monthly household income of CNY 4,000 (see Table 1).

**Table 1:** Sample characteristics by gender.

Variable	Total (*N* = 1,276)	Female (*n* = 639)	Male (*n* = 637)
Gender, *n* (%)	1,276 (100.0)	639 (50.1)	637 (49.9)
Age			
18–19 years, *n* (%)	487 (38.2)	219 (34.3)	268 (42.1)
20–21 years, *n* (%)	630 (49.3)	332 (52.0)	298 (46.8)
22 years and above, *n* (%)	159 (12.5)	88 (13.7)	71 (11.1)
*M (SD)*	19.94 (1.34)	20.04 (1.32)	19.83 (1.36)
Age of first smartphone ownership			
6–10 years, *n* (%)	61 (4.8)	31 (4.9)	30 (4.7)
11–15 years, *n* (%)	535 (41.9)	273 (42.7)	262 (41.1)
16–21 years, *n* (%)	680 (53.3)	335 (52.4)	345 (54.2)
*M (SD)*	15.39 (2.51)	15.30 (2.50)	15.49 (2.52)
Monthly household income			
< *CNY* 2,683, *n* (%)	309 (24.2)	168 (26.3)	141 (22.1)
*CNY* 2,684-4,184, *n* (%)	452 (35.4)	229 (35.8)	223 (35.1)
*CNY* 4,185-7,920, *n* (%)	414 (32.5)	201 (31.5)	213 (33.4)
≥ *CNY* 7,921, *n* (%)	101 (7.9)	41 (6.4)	60 (9.4)
*Mdn*	4,000	4,000	4,000

M, mean; SD, standard deviation; Mdn, median; CNY, Chinese yuan.

For the key study variables, male students reported slightly higher mean scores for smartphone addiction, reinterpretation strategy, suppression strategy, and mental health symptoms than female students. Observed minimum and maximum values indicated adequate variability across all constructs (see Table 2).

**Table 2:** Descriptive statistics for key study variables by gender.

Variable	Gender	*n*	*M*	*SD*	*Min*	*Max*
Smartphone addiction	Female	639	37.33	4.39	33	51
Smartphone addiction	Male	637	38.50	4.97	31	51
Reinterpretation strategy	Female	639	4.85	0.81	3	7
Reinterpretation strategy	Male	637	5.06	0.91	3	7
Suppression strategy	Female	639	3.79	1.05	1	7
Suppression strategy	Male	637	4.41	1.14	1	7
Mental health symptoms	Female	639	2.19	0.82	1	5
Mental health symptoms	Male	637	2.53	0.94	1	5

M, mean; SD, standard deviation.

Pearson correlation analyses showed that smartphone addiction was positively correlated with reinterpretation strategy, *r* = 0.283, *p* < 0.001; suppression strategy, *r* = 0.324, *p* < 0.001; and mental health symptoms, *r* = 0.609, *p* < 0.001. Suppression strategy was positively correlated with mental health symptoms, *r* = 0.422, *p* < 0.001. Reinterpretation strategy showed a weak positive bivariate correlation with mental health symptoms, *r* = 0.060, *p* < 0.05. These bivariate results should be distinguished from the structural model estimates, in which reinterpretation strategy showed a negative association with mental health symptoms after other predictors were modelled simultaneously (see Table 3).

**Table 3:** Pearson correlations among study variables.

Variable	*M (SD)*	1	2	3	4
1. Smartphone addiction	37.92 (4.72)	–			
2. Reinterpretation strategy	4.96 (0.87)	0.283***	–		
3. Suppression strategy	4.10 (1.14)	0.324***	0.334***	–	
4. Mental health symptoms	2.36 (0.89)	0.609***	0.060*	0.422***	–

*p < 0.05, ***p < 0.001. Correlations are bivariate Pearson correlations.

### Measurement model evaluation

The reflective measurement model was evaluated before testing the structural relationships. Internal consistency reliability was examined using Cronbach’s alpha and composite reliability (CR; Hair et al., 2019). All constructs showed acceptable reliability, with CR values between 0.790 and 0.949 (see Table 4).

**Table 4:** Reflective measurement model results.

Construct/item	Loading	*α*	*CR*	*AVE*
Mental health symptoms		0.935	0.949	0.685
PSD1	0.753			
PSD2	0.783			
PSD3	0.831			
PSD4	0.837			
PSD5	0.833			
PSD6	0.855			
PSD7	0.840			
PSD8	0.871			
PSD9	0.852			
PSD10	0.812			
Smartphone addiction		0.896	0.911	0.556
SA1	0.752			
SA2	0.781			
SA3	0.741			
SA4	0.761			
SA5	0.556			
SA6	0.801			
SA7	0.763			
SA8	0.758			
SA9	0.785			
SA10	0.734			
Reinterpretation strategy		0.878	0.871	0.606
RA1	0.774			
RA2	0.765			
RA3	0.725			
RA4	0.793			
RA5	0.817			
RA6	0.794			
Suppression strategy		0.842	0.790	0.615
IH1	0.800			
IH2	0.706			
IH3	0.821			
IH4	0.805			

α, Cronbach’s alpha; CR, composite reliability; AVE, average variance extracted; PSD, mental health symptoms; SA, smartphone addiction; RA, reinterpretation strategy; IH, suppression strategy.

Convergent validity was assessed using average variance extracted (AVE). The AVE values for all constructs were above the recommended 0.50 benchmark, suggesting that the constructs captured sufficient variance from their indicators. Indicator loadings were generally acceptable, further supporting convergent validity.

Discriminant validity was examined using the heterotrait–monotrait ratio of correlations (HTMT). Because all HTMT values were below 0.90, the constructs were considered empirically distinguishable. However, the HTMT value between smartphone addiction scores and mental health symptoms was relatively high (HTMT = 0.817), suggesting substantial conceptual and empirical overlap between these constructs. Therefore, although the discriminant validity criterion was met, the association between these two constructs should be interpreted with caution (see Table 5).

**Table 5:** Discriminant validity based on HTMT ratios.

Variable	1	2	3	4
1. Smartphone addiction	–			
2. Suppression strategy	0.417	–		
3. Reinterpretation strategy	0.243	0.400	–	
4. Mental health symptoms	0.817	0.484	0.076	–

HTMT, heterotrait-monotrait ratio. Discriminant validity is supported when HTMT < 0.90. The HTMT value between smartphone addiction and mental health symptoms was relatively high (0.817), suggesting conceptual overlap that should be considered when interpreting these constructs.

### Structural model results

Collinearity was evaluated before examining the structural coefficients. All VIF values fell between 1.000 and 1.244, which was below the recommended thresholds of 3.3 and 5.0, suggesting that multicollinearity was not problematic (Hair et al., 2019; Kock, 2015).

The analysis of direct effects indicated a strong positive association between smartphone addiction and mental health symptoms, *β* = 0.748, *t* = 55.524, *p* < 0.001, 95% *CI* [0.722, 0.775]. Smartphone addiction was also significantly and positively associated with suppression strategy, *β* = 0.357, *t* = 12.075, *p* < 0.001, 95% *CI* [0.299, 0.414], and reinterpretation strategy, *β* = 0.214, *t* = 7.230, *p* < 0.001, 95% *CI* [0.158, 0.276]. Suppression strategy was positively associated with mental health symptoms, *β* = 0.205, *t* = 10.241, *p* < 0.001, 95% *CI* [0.166, 0.245], whereas reinterpretation strategy was negatively associated with mental health symptoms, *β* = −0.166, *t* = 9.269, *p* < 0.001, 95% *CI* [−0.202, −0.131] (see Table 6).

**Table 6:** Results of PLS-SEM analysis.

Path	*β*	*t*	*p*	*95% CI*	*VIF*
Direct associations					
SA → PSD	0.748	55.524	< 0.001	[0.722, 0.775]	1.160
IH → PSD	0.205	10.241	< 0.001	[0.166, 0.245]	1.244
RA → PSD	−0.166	9.269	< 0.001	[−0.202, −0.131]	1.138
SA → IH	0.357	12.075	< 0.001	[0.299, 0.414]	1.000
SA → RA	0.214	7.230	< 0.001	[0.158, 0.276]	1.000
Indirect associations					
SA → IH → PSD	0.073	7.678	< 0.001	[0.055, 0.092]	–
SA → RA → PSD	−0.036	5.774	< 0.001	[−0.049, −0.025]	–

SA, smartphone addiction; RA, reinterpretation strategy; IH, suppression strategy; PSD, mental health symptoms; CI, confidence interval; VIF, variance inflation factor.

Effect size estimates indicated a large association between smartphone addiction and mental health symptoms, *f^2^* = 0.533. Small effect sizes were observed for the associations from smartphone addiction to suppression strategy, *f^2^* = 0.119, and from suppression strategy to mental health symptoms, *f^2^* = 0.139. The associations involving reinterpretation strategy were smaller, with *f^2^* = 0.088 for smartphone addiction to reinterpretation strategy and *f^2^* = 0.066 for reinterpretation strategy to mental health symptoms.

### Statistical indirect associations

Indirect associations were examined using bootstrapping procedures. The indirect association from smartphone addiction to mental health symptoms through suppression strategy was positive and statistically significant, *β* = 0.073, *t* = 7.678, *p* < 0.001, 95% *CI* [0.055, 0.092]. In contrast, the indirect association through reinterpretation strategy was negative and statistically significant, *β* = −0.036, *t* = 5.774, *p* < 0.001, 95% *CI* [−0.049, −0.025]. These findings indicate that suppression and reinterpretation strategies were involved in statistically significant indirect associations in opposite directions. Because all variables were measured at a single time point, the estimated indirect effects cannot establish causal mediation and should be interpreted only as associational evidence.

### Model explanatory power and predictive relevance

R^2^ was examined to determine how much variance was explained in the endogenous constructs. Predictive relevance was assessed separately using *Q^2^*_predict values generated by the PLSpredict procedure (Shmueli et al., 2019). Reporting *Q^2^*_predict is appropriate in PLS-SEM because it provides information about the model’s ability to predict endogenous construct indicators beyond in-sample explanatory capacity.

The structural model explained a substantial proportion of variance in mental health symptoms, *R^2^* = 0.662, and a modest proportion of variance in suppression strategy, *R^2^* = 0.127. The variance explained in the reinterpretation strategy was relatively limited, *R^2^* = 0.045. The *Q^2^*_predict values were 0.616 for mental health symptoms, 0.125 for suppression strategy, and 0.043 for reinterpretation strategy. All values were greater than zero, indicating acceptable predictive relevance, with comparatively stronger predictive performance for mental health symptoms.

### Gender-based multigroup analysis

#### Measurement invariance assessment

Measurement invariance across gender was assessed with the MICOM permutation procedure before conducting multigroup comparisons. As reported in Table 7, configural invariance was established because identical indicators, data-treatment procedures, and algorithm settings were used for male and female students. Compositional invariance was likewise supported for all constructs, with original correlations of 1.000 that were not lower than the corresponding 5.0% permutation quantiles. However, equality of composite means and variances was not observed for every construct; thus, full measurement invariance was not achieved. Because the first two MICOM steps were satisfied, partial measurement invariance was established, allowing meaningful comparison of structural path coefficients across gender groups.

**Table 7:** Results of measurement invariance assessment using the MICOM permutation procedure.

Construct	Configural invariance (Step 1)	Original correlation	5.0% quantile	Compositional invariance (Step 2)	Original difference	95% CI for mean difference	Original difference	*95% CI* for variance difference	Full measurement invariance
PSD	Yes	1.000	1.000	Yes	0.378	[−0.100, 0.100]	0.273	[−0.139, 0.134]	No
IH	Yes	1.000	1.000	Yes	0.550	[−0.116, 0.105]	0.173	[−0.159, 0.170]	No
RA	Yes	1.000	1.000	Yes	0.225	[−0.111, 0.113]	0.255	[−0.154, 0.142]	No
SA	Yes	1.000	1.000	Yes	0.251	[−0.104, 0.110]	0.212	[−0.152, 0.145]	No

PSD, mental health symptoms; IH, suppression strategy; RA, reinterpretation strategy; SA, smartphone addiction. MICOM, measurement invariance of composite models; CI, confidence interval. Compositional invariance is established when the original correlation is not lower than the 5.0% permutation quantile. Because configural and compositional invariance were established for all constructs, partial measurement invariance was supported. Full measurement invariance was not established because equality of composite means and/or variances was not supported.

#### Multigroup structural model results

**Figure 2:**
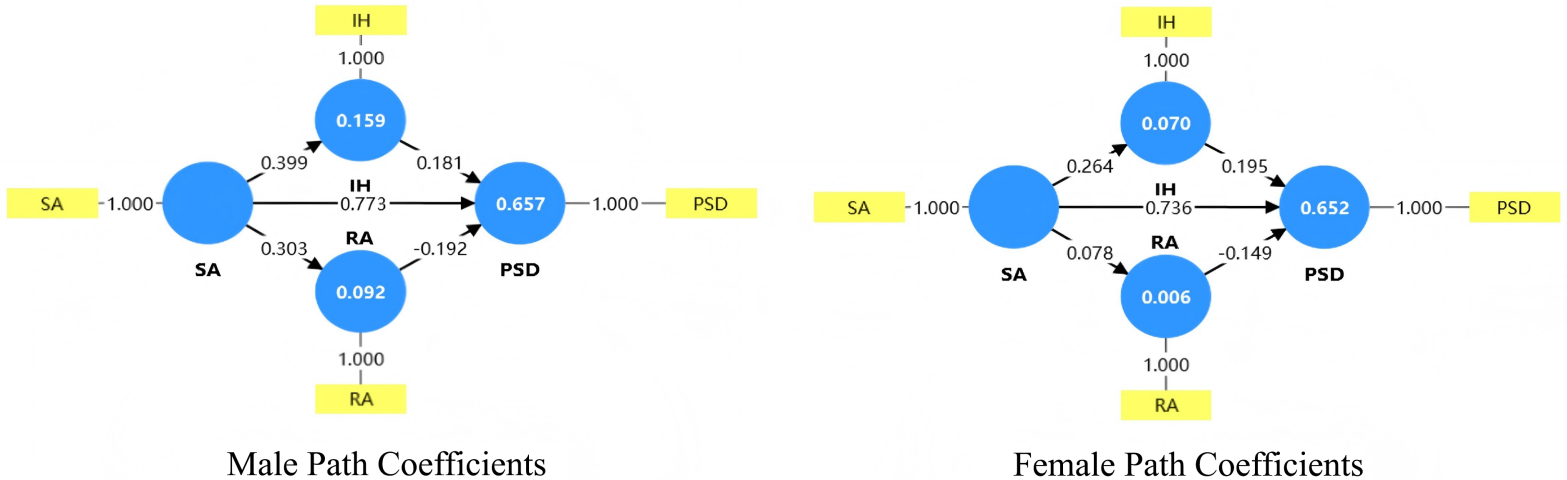
Group-specific structural model by student gender (standardized path coefficients).

Multigroup analysis results are presented in Table 8, with standardized path coefficients illustrated in Figure 2. The overall pattern of structural associations was similar for male and female students, although several paths differed in magnitude. Because male and female participants were included using different SAS-SV screening thresholds, the multigroup findings should be interpreted as gender-group differences within the present screening procedure rather than as direct evidence of gender-based causal differences.

**Table 8:** Gender-based multigroup analysis.

Path	*β* Male	*β* Female	*t* Male	*t* Female	*p* Male	*p* Female	*Δβ*	*p* Difference
Direct associations								
SA → PSD	0.773	0.736	33.348	40.372	< 0.001	< 0.001	0.037	0.089
IH → PSD	0.181	0.195	5.379	7.568	< 0.001	< 0.001	−0.014	0.336
RA → PSD	−0.192	−0.149	7.298	5.851	< 0.001	< 0.001	−0.043	0.100
SA → IH	0.399	0.264	10.298	5.598	< 0.001	< 0.001	0.135	0.009
SA → RA	0.303	0.078	7.662	1.716	< 0.001	0.086	0.225	< 0.001
Indirect associations								
SA → RA → PSD	−0.058	−0.012	5.285	1.642	< 0.001	0.101	−0.047	< 0.001
SA → IH → PSD	0.072	0.051	4.703	4.497	< 0.001	< 0.001	0.021	0.152

SA, smartphone addiction; RA, reinterpretation strategy; IH, suppression strategy; PSD, mental health symptoms; Δβ, male–female difference in standardized coefficients.

Smartphone addiction was significantly and positively related to mental health symptoms among both male students, *β* = 0.773, *t* = 33.348, *p* < 0.001, and female students, *β* = 0.736, *t* = 40.372, *p* < 0.001. Although the coefficient was slightly larger for males, the gender difference was not statistically significant, *Δβ* = 0.037, *p* = 0.089.

Suppression strategy was positively associated with mental health symptoms among males, *β* = 0.181, *t* = 5.379, *p* < 0.001, and females, *β* = 0.195, *t* = 7.568, *p* < 0.001. The gender difference for this pathway was not significant, *Δβ* = −0.014, *p* = 0.336. Reinterpretation strategy was significantly and negatively related to mental health symptoms among males, *β* = −0.192, *t* = 7.298, *p* < 0.001, and females, *β* = −0.149, *t* = 5.851, *p* < 0.001. However, the male–female difference in this association was not statistically significant, *Δβ* = −0.043, *p* = 0.100.

Gender differences emerged for the paths linking smartphone addiction to the coping strategies. The association between smartphone addiction and suppression strategy was stronger among male students, *β* = 0.399, *t* = 10.298, *p* < 0.001, than among female students, *β* = 0.264, *t* = 5.598, *p* < 0.001, with a significant between-group difference, *Δβ* = 0.135, *p* = 0.009. The association between smartphone addiction and reinterpretation strategy was also stronger among male students, *β* = 0.303, *t* = 7.662, *p* < 0.001, than among female students, *β* = 0.078, *t* = 1.716, *p* = 0.086, with a significant between-group difference, *Δβ* = 0.225, *p* < 0.001.

#### Gender differences in statistical indirect associations

The indirect association through suppression strategy was positive and statistically significant for both male students, *β* = 0.072, *t* = 4.703, *p* < 0.001, and female students, *β* = 0.051, *t* = 4.497, *p* < 0.001. The between-group difference was not statistically significant, *Δβ* = 0.021, *p* = 0.152.

A gender-specific pattern was observed for the indirect association through the reinterpretation strategy. Among male students, smartphone addiction scores were indirectly and negatively associated with mental health symptoms through reinterpretation strategy, *β* = −0.058, *t* = 5.285, *p* < 0.001. Among female students, this indirect association was not statistically significant, *β* = −0.012, *t* = 1.642, *p* = 0.101. The between-group difference was statistically significant, *Δβ* = −0.047, *p* < 0.001. These findings indicate that the statistical indirect association involving the reinterpretation strategy was more pronounced among male students than among female students within the present screening-based sample.

## Discussion

Grounded in Stress-Coping Theory, this study examined cross-sectional associations linking smartphone addiction scores, emotional coping strategies, mental health symptoms, and gender among Chinese university students who met the SAS-SV screening threshold. The findings showed that smartphone addiction scores were strongly and positively associated with mental health symptoms. The suppression strategy was positively associated with mental health symptoms, whereas the reinterpretation strategy was negatively associated with mental health symptoms in the structural model. Gender did not significantly differentiate the direct association between smartphone addiction scores and mental health symptoms, but it differentiated the indirect association involving the reinterpretation strategy.

### Smartphone addiction and mental health symptoms

Consistent with prior empirical findings, smartphone addiction scores showed a robust positive association with mental health symptoms (Luo et al., 2025; Lei et al., 2020; Feng and Dou, 2024). This finding is consistent with prior studies linking addiction-like or problematic smartphone use to poorer psychological adjustment, but it extends this literature by focusing specifically on students who met established SAS-SV screening criteria rather than on general smartphone use in unselected student samples. In this respect, the present findings provide more targeted evidence for a screening-positive subgroup within Chinese higher education. This result supports the view that elevated addiction-like smartphone use is closely linked to poorer psychological adjustment among university students. Within a stress-coping framework, excessive smartphone use may be understood as a digital stress context involving constant connectivity, information overload, disrupted sleep, and frequent interruptions. These stress-related features may be associated with anxiety, depressive symptoms, and emotional exhaustion.

The relatively strong association observed in this study suggests that smartphone addiction scores are closely linked to mental health symptoms among Chinese university students. Within competitive academic settings, where digital technologies are embedded in learning and social interaction, problematic smartphone use may overlap with academic demands and social stressors. However, because the data were cross-sectional, the temporal ordering of these relationships remains unclear. It is therefore not possible to determine whether elevated addiction-like smartphone use precedes mental health symptoms, whether psychological distress increases susceptibility to problematic smartphone use, or whether both arise from shared underlying risk factors.

### Emotional coping strategies as indirect correlates

Beyond the direct association, the findings identify emotional coping strategies as important correlates linking smartphone addiction scores with mental health symptoms. The structural model showed opposite associations for the two emotion regulation strategies. The suppression strategy was positively associated with mental health symptoms, whereas the reinterpretation strategy was negatively associated with these symptoms. This finding is consistent with prior emotion regulation research showing that suppression is typically associated with poorer psychological adjustment, while cognitive reinterpretation or reappraisal is more often related to adaptive psychological functioning (Chen et al., 2025; Gross and John, 2003).

The significant indirect associations suggest that students with higher smartphone addiction scores tended to report different patterns of emotional coping, and these coping strategies were, in turn, statistically linked to mental health symptoms. Suppression may reflect an avoidant response pattern in which emotional expression is inhibited without addressing the underlying stressor. In contrast, reinterpretation may reflect a more adaptive cognitive strategy that is inversely associated with mental health symptoms. Importantly, these findings should be interpreted as cross-sectional statistical associations rather than evidence that smartphone addiction causes changes in emotional coping or that coping strategies causally transmit their effects to mental health symptoms.

The variance explained in the reinterpretation strategy was relatively modest, suggesting that reinterpretation is likely shaped by a broader set of factors beyond smartphone addiction, including dispositional traits, emotional competencies, family context, and social resources. This interpretation aligns with theoretical perspectives that view reinterpretation as a regulatory strategy influenced by both individual and contextual characteristics (John and Gross, 2004).

### Gender differences in indirect associations

This study examined gender-group differences in the structural associations, but these differences should be interpreted cautiously. Smartphone addiction scores were positively associated with mental health symptoms among both male and female students, and the between-group difference in this direct association was not statistically significant. This finding suggests that the association between smartphone addiction scores and mental health symptoms is not confined to one gender group.

Gender-group differences were observed in the indirect association involving the reinterpretation strategy. Specifically, the indirect association through the reinterpretation strategy was statistically significant among male students but not among female students. This result is consistent with prior evidence that gender may shape emotion regulation patterns and coping-related psychological adjustment (Salerno et al., 2022; Cholankeril et al., 2023; Preston et al., 2022). One possible interpretation is that reinterpretation may be particularly relevant to mental health symptoms among male students with elevated addiction-like smartphone use. However, because this finding is based on cross-sectional multigroup analysis and on groups defined using gender-specific SAS-SV cutoff values, it should be treated as a gender-differentiated statistical association that requires replication in longitudinal research and in samples defined using common or continuously modelled smartphone-use scores.

In contrast, the suppression strategy showed a positive indirect association among both male and female students, and the between-group difference was not significant. This suggests that suppression may represent a broadly maladaptive emotional coping correlate across gender groups. The absence of gender difference in the direct association also cautions against assuming that one gender is inherently more vulnerable to smartphone-related mental health symptoms. Instead, the findings support the value of examining specific psychological processes and gender-conditioned associations while recognizing that the observed multigroup differences cannot be attributed directly to gender alone.

### Theoretical contributions

The present study makes several theoretical contributions. First, it extends research on smartphone addiction scores and mental health by examining both direct and indirect statistical associations involving emotional coping strategies. By modelling reinterpretation and suppression strategies simultaneously, the study provides a more differentiated account of how adaptive and maladaptive coping strategies are associated with mental health symptoms among students with elevated addiction-like smartphone use.

Second, the integration of gender-based multigroup analysis within a stress-coping framework contributes to research on individual differences in technology-related mental health. Rather than treating gender only as a demographic control variable, this study examined whether structural associations differed between male and female students. The findings indicate that gender may be more relevant to specific indirect associations than to the direct association between smartphone addiction scores and mental health symptoms, although the interpretation of gender-group differences must consider the gender-specific SAS-SV screening thresholds used to construct the analytical sample.

Third, PLS-SEM was used as an acceptable variance-based SEM approach to estimate measurement quality, structural associations, indirect statistical associations, predictive relevance, and gender-based multigroup differences (Hair et al., 2019; Shmueli et al., 2019). This analytical choice should be interpreted as one defensible approach rather than as evidence that PLS-SEM was uniquely required. Future research using covariance-based SEM, longitudinal SEM, or cross-lagged panel models would provide stronger evidence regarding global model fit, temporal ordering, and directionality.

### Practical implications

The results have practical relevance for mental health promotion in higher education settings. Because smartphone addiction scores were strongly associated with mental health symptoms, identifying students with elevated addiction-like smartphone use at an early stage remains necessary. However, intervention programs should not be limited to reducing screen time or controlling usage frequency. The findings suggest that students’ emotional coping strategies should also be addressed in prevention, psychoeducation, and counselling programs.

Programs designed to strengthen adaptive reinterpretation and reduce reliance on suppression may be useful components of student mental health services, stress management workshops, and digital well-being initiatives. The observed gender-specific pattern further suggests that intervention design may benefit from sensitivity to gender differences in coping-related associations. However, because the gender groups were defined using different SAS-SV screening thresholds, these implications should remain tentative. These implications should also be interpreted cautiously because the present findings are correlational and do not establish intervention effects.

### Limitations and future directions

Several limitations should be noted. First, because the study used a cross-sectional design, the findings cannot establish causality, temporal sequence, or directionality. Longitudinal, cross-lagged, and experimental research is needed to determine whether elevated addiction-like smartphone use precedes mental health symptoms, whether psychological difficulties increase vulnerability to problematic smartphone use, or whether reciprocal associations are present.

Second, all constructs were measured through self-report questionnaires, which may be affected by recall bias, social desirability, and common method variance. Although Harman’s single-factor analysis indicated that the first factor accounted for less than 50% of the total variance, the result approached the conventional benchmark. Therefore, this test cannot fully address concerns about common method variance. Future studies should incorporate multi-informant data, behavioral indicators, passive sensing data, or temporal separation of measurements.

Third, the sample comprised Chinese university students who met the SAS-SV screening threshold for elevated addiction-like smartphone use. This focus was appropriate for the study aim but may limit generalizability to students without elevated smartphone-use problems or to populations in other cultural contexts. The SAS-SV cutoff identifies a screening-positive group rather than a formal clinical diagnosis. Smartphone addiction is not formally recognized as a diagnostic disorder in the DSM-5, and the present findings should not be interpreted as evidence concerning clinically diagnosed smartphone addiction. Care is therefore needed to avoid stigmatizing students with high-frequency or problematic smartphone use.

Fourth, the validity of the SAS-SV screening thresholds should be interpreted cautiously. The gender-specific cutoff values proposed by Kwon et al. (2013) were used to define the analytical sample, and the Chinese version of the SAS-SV has shown acceptable psychometric properties among Chinese college students (Zhao et al., 2022). However, evidence of reliability and factorial validity does not necessarily establish that the same cutoff scores have equivalent classification validity across cultural or educational populations. Future research should further examine measurement invariance, ROC-based cutoff validity, and clinical validation of the SAS-SV among Chinese university students.

Fifth, the use of gender-specific SAS-SV thresholds may have affected group composition and range restriction in the gender-based multigroup analysis. Male students with SAS-SV scores of 31–32 were included, whereas female students with comparable scores were not included. Consequently, the observed gender-group differences may partly reflect differences in screening-based group composition rather than gender itself. The gender-based MGA results therefore cannot be attributed directly to gender alone and should be interpreted as preliminary associations within the present screening procedure.

Sixth, although all HTMT values met the recommended discriminant validity criterion, the HTMT value between smartphone addiction scores and mental health symptoms was relatively high (0.817). This result suggests conceptual and empirical overlap between elevated addiction-like smartphone use and psychological distress symptoms. Future studies should further examine the discriminant validity of these constructs using alternative measurement models and longitudinal designs.

Finally, PLS-SEM was used as an acceptable variance-based SEM approach for the present analytical aims, but it was not the only possible SEM framework. Because the model involved theoretically specified associations among reflective constructs and because the sample size was large, future research could use CB-SEM to evaluate global model fit and compare alternative theoretical models. In addition, the exclusion of problem-focused coping restricts the scope of the coping model; future studies should include active problem solving, planning, and instrumental support to clarify how problem-focused and emotion-focused coping strategies jointly relate to elevated addiction-like smartphone use and mental health symptoms. Multi-wave designs would be particularly useful for testing whether emotional coping strategies operate as temporal correlates over time.

## Conclusion

This study provides evidence of meaningful cross-sectional associations among smartphone addiction scores, emotional coping strategies, and mental health symptoms among Chinese university students. By conceptually distinguishing elevated addiction-like smartphone use from the broader construct of problematic smartphone use and from clinical diagnosis, the study clarifies that its findings apply to students who met SAS-SV screening thresholds. Suppression strategy showed a positive indirect statistical association, whereas reinterpretation strategy showed a negative indirect statistical association, indicating that different emotional coping strategies are linked to mental health symptoms in distinct ways. Gender-group differences were observed in the reinterpretation-related indirect association, but these differences should be interpreted cautiously because gender-specific SAS-SV cutoff values may have influenced group composition. These findings contribute to stress-coping theory by extending it to addiction-like smartphone use, while also underscoring the need for longitudinal and experimental studies to establish temporal ordering and causal direction.

## Data Availability

The datasets analyzed in this study are available from the corresponding author upon reasonable request, subject to applicable ethical and institutional data-sharing restrictions.
